# Heterologous Expression of the Melatonin-Related Gene *HIOMT* Improves Salt Tolerance in *Malus domestica*

**DOI:** 10.3390/ijms222212425

**Published:** 2021-11-17

**Authors:** Kexin Tan, Jiangzhu Zheng, Cheng Liu, Xianghan Liu, Xiaomin Liu, Tengteng Gao, Xinyang Song, Zhiwei Wei, Fengwang Ma, Chao Li

**Affiliations:** State Key Laboratory of Crop Stress Biology for Arid Areas/Shaanxi Key Laboratory of Apple, College of Horticulture, Northwest A & F University, Yangling, Xianyang 712100, China; kexin163_2020@163.com (K.T.); jiayouzjz@163.com (J.Z.); liucheng175303479@163.com (C.L.); baimoliuxianghan@163.com (X.L.); lxm0603@nwafu.edu.cn (X.L.); 15829268875@163.com (T.G.); songlina12345@163.com (X.S.); weizhiwei89@126.com (Z.W.)

**Keywords:** hydroxyindole-O-methyltransferase gene, melatonin, ROS, ABA, ion homeostasis, amino acids

## Abstract

Melatonin, a widely known indoleamine molecule that mediates various animal and plant physiological processes, is formed from N-acetyl serotonin via N-acetylserotonin methyltransferase (ASMT). ASMT is an enzyme that catalyzes melatonin synthesis in plants in the rate-determining step and is homologous to hydroxyindole-O-methyltransferase (HIOMT) melatonin synthase in animals. To date, little is known about the effect of *HIOMT* on salinity in apple plants. Here, we explored the melatonin physiological function in the salinity condition response by heterologous expressing the homologous human *HIOMT* gene in apple plants. We discovered that the expression of melatonin-related gene (*MdASMT*) in apple plants was induced by salinity. Most notably, compared with the wild type, three transgenic lines indicated higher melatonin levels, and the heterologous expression of *HIOMT* enhanced the expression of melatonin synthesis genes. The transgenic lines showed reduced salt damage symptoms, lower relative electrolyte leakage, and less total chlorophyll loss from leaves under salt stress. Meanwhile, through enhanced activity of antioxidant enzymes, transgenic lines decreased the reactive oxygen species accumulation, downregulated the expression of the abscisic acid synthesis gene (*MdNCED3*), accordingly reducing the accumulation of abscisic acid under salt stress. Both mechanisms regulated morphological changes in the stomata synergistically, thereby mitigating damage to the plants’ photosynthetic ability. In addition, transgenic plants also effectively stabilized their ion balance, raised the expression of salt stress–related genes, as well as alleviated osmotic stress through changes in amino acid metabolism. In summary, heterologous expression of *HIOMT* improved the adaptation of apple leaves to salt stress, primarily by increasing melatonin concentration, maintaining a high photosynthetic capacity, reducing reactive oxygen species accumulation, and maintaining normal ion homeostasis.

## 1. Introduction

Apple (*Malus domestica* Borkh.) is one of the main fruits in the world, the cultivation in China is mainly concentrated in bohai Bay and Loess Plateau, which are two dominant producing areas. Soil salinization in these areas is one of the main obstacles to the expansion of apple eugenic cultivation areas [1]. The sustainable development of apple industry urgently needs the support of apple salt tolerance strategy and technology.

Soil salinity is a progressively serious issue worldwide, as it hinders plant development and causes yield losses [1]. The detrimental influence of NaCl stress on apple plants has two primary aspects [2]. First, the excessive sodium ions accumulation results in a significant decrease in the effective use of water [3], second, Na^+^ and Cl^−^ ions toxic effects lead to ion imbalances [4]. The resulting disturbances in most physiological processes include growth inhibition, reduction in photosynthetic capacity, destruction of membranes, changes in enzymatic activities, and ionic imbalances [5]. These phenomena emphasize the urgency of improving apple plants salt stress tolerance.

Photosynthesis supplies the energy source for plant normal growth, while the photosynthetic apparatus are sensitive to external stress [6]. Salinity stress reduces leaf photosynthetic capacity through both stomatal and non-stomatal mechanisms [7]. Stomatal limitations result from exposure to high salt concentrations; plants become dehydrated due to a reduction of osmotic potential and turgor pressure, causing a decrease in stomatal aperture and reducing CO_2_ assimilation capacity, which in turn reduce net photosynthesis [8]. Non-stomatal limitations are related to damage to photosystem II (PSII), CO_2_ assimilation rate, and electron transport chain (ETC) efficiency [9].

Plants take up specialized approaches to cope with high salt concentrations. Reactive oxygen species (ROS) production is one of the initial reactions caused by salinity [10], and excessive ROS accumulation can initiate lipid peroxidation processes and increase the concentration of malondialdehyde (MDA), ultimately increasing the plasma membrane permeability, causing intracellular electrolytes leakage [11]. To mitigate ROS damage to various tissues, plants trigger salt-induced antioxidant systems, increasing the activity of superoxide dismutase (SOD), peroxidase (POD), ascorbate peroxidase (APX), and related enzymes. Together, these antioxidant enzymes interact to scavenge ROS and improve plant salt tolerance [12]. Studies have shown that ROS accumulation can act as a positive modifier to mediate abscisic acid (ABA) signal and response. As an abiotic stress phytohormone, ABA has a central function in salt stress defense [13]. Under salt stress, the CLAVATA3/ESR-RELATED 25 (CLE25) peptide may be secreted and moved to the shoots where it is identified by the receptors BARELY ANY MERISTEM 1 (BAM1) and BAM3, thus promoting the expression of the ABA biosynthesis gene *NCED3* [14]. This is one of the mechanisms of plant root from the dehydration signal under salinity condition.

The sequestration of toxic ions and the biosynthesis of osmotic substances in plants under salt stress are related to improved osmotic stress tolerance [15]. The intracellular ion balance does not allow toxic ions to accumulate in the cytoplasm and requires net Na^+^ and Cl^−^ uptake and subsequent vacuolar separation [16]. Appropriate Na^+^/K^+^ homeostasis maintains the normal physiological metabolism of plants [17], and the absorption, biosynthesis, and transformation of amino acids under stress can alleviate associated damage [18]. As a crucial mechanism for plants response to salt stress, SOS transports excess Na^+^ to the extracellular space using the plasma membrane proton gradient [15,19]. NHX family members act on the tonoplast membrane, and the activity of NHX1, NHX2, and NHX4 hinges on the proton gradient to achieve Na^+^ compartmentalization [20]. The activity of low-affinity K channel (AKT) is also closely associated with salt tolerance. Free amino acid concentrations also respond sensitively to salinity stress. For example, it is common that physiological response of different plants accumulating Pro under salinity condition [21]. Salt stress results in upregulation of genes in the aromatic amino acid (AAA) biosynthetic pathway, increasing the AAA concentration and the production of related secondary metabolites such as auxins, alkaloids, and flavonoids, thereby improving abiotic stress tolerance [22].

Multitude studies have demonstrated that melatonin is an indole molecule with multiple regulatory functions that has a considerable role in protecting plants from abiotic stresses, including salinity–alkalinity, drought, temperature extremes, oxidative stress, and UV radiation [23,24,25,26,27]. The biosynthetic pathway of plant melatonin is well characterized. First, tryptophan decarboxylase (TDC) promotes the conversion of tryptophan to tryptamine. Then serotonin is produced by the hydroxylation reaction of tryptophan 5-hydroxylase (T5H), and finally, arylalkylamine-N-acetyltransferase (AANAT) and N-acetylserotonin methyltransferase (ASMT) catalyze the synthesis of plant melatonin [26,27]. At present, there are abundant researches show that exogenous melatonin can improve plant resistance under salt-alkali stress [24,25,26]. Among them, Li et al. [24] found that exogenous melatonin by enhancing the activity of antioxidant enzymes and stabilizing the ion balance to enhance the salt tolerance of *Malus hupehensis*. Recently, several studies have documented the physiological functions of melatonin in plants with heterologous overexpression of melatonin biosynthetic genes [28]. For example, Huang et al. [29] demonstrated that transfer ovine *AANAT*, *HIOMT* genes into switchgrass improved its growth and salinity endurance under salt stress. Droxyindole-O-methyltransferase (*HIOMT*), encodes the last enzyme catalyzes the synthesis of animal melatonin, is a homolog of *ASMT* in apple plants, plays a rate-limiting role in the melatonin synthesis pathway. Its function is to convert N-acetyl serotonin into melatonin [30,31]. In our research team, Liu et al. [31] previously found that ectopic expression *AANAT* and *HIOMT* in apple plants mainly by scavenging reactive oxygen species and increasing total phenolic content to improve resistance to UV-B stress. Although the regulation of *HIOMT* on the UV-B stress response of apples has been described, the mechanism by which *HIOMT* acts in *Malus domestica* under salinity condition has not yet been known clearly. Here, we used hydroponic methods to explore in detail the mechanisms by which *HIOMT* heterologous expression impacts the salinity response of *Malus domestica*.

## 2. Results

### 2.1. Heterologous Expression of HIOMT Improved Apple Salt Stress Tolerance and Increased Melatonin Concentration in Apple Leaves

**Figure 1:**
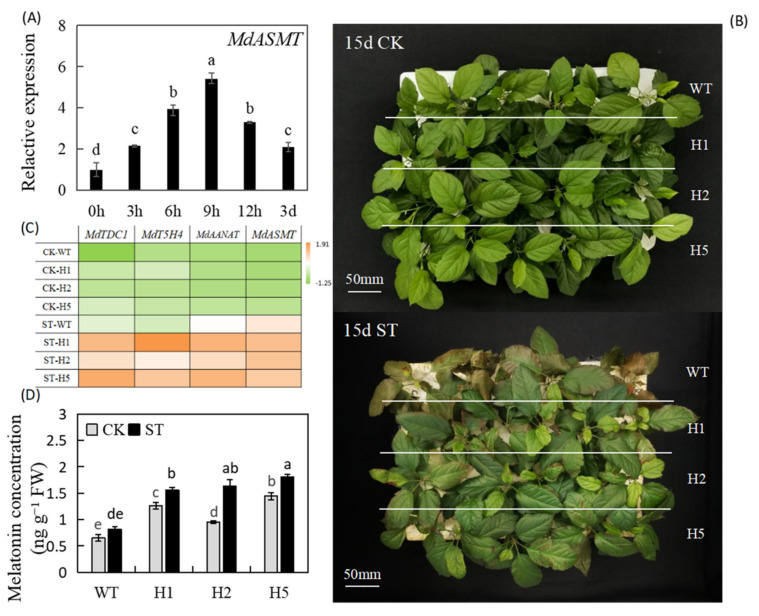
Heterologous expression of *HIOMT* confers enhanced salinity tolerance to apple. (**A**) The expression of *MdASMT* after salt treatment in GL-3 apple; (**B**) phenotypes of WT and transgenic lines under control and 120 mM NaCl condition. Bars: 50 mm; (**C**) The expression of MT-related synthesis genes under control and salinity conditions; (**D**) the concentration of melatonin after salinity treatment. Values are noted as means of 3 replicates ± SE. Based on Tukey’ s multi-range test (*p* < 0.05), not the same lowercase letters were used to represent statistically significant differences. WT, wild type. H1, heterologous expression of *HIOMT* line 1. H2, heterologous expression of *HIOMT* line 2. H5, heterologous expression of *HIOMT* line 5. CK, control group. ST, salt stress group.

In GL-3 (*Malus domestica* Borkh.) plants, salinity induced the heterologous expression of the *HIOMT* homolog *MdASMT*; its expression was highest at 9 h, when it was upregulated 5.39-fold relative to unstressed controls (Figure 1A). To explore the possible physiological role of *HIOMT* in the apple salinity response, we exposed heterologous expression of the *HIOMT* lines previously obtained to salt stress under hydroponic conditions [31]. No notable deviations between the transgenic lines and the wild type (WT) under normal nutritional conditions. By contrast, all genotypes exhibited damage after NaCl treatment for 15 d. Nonetheless, all leaves of the WT exhibited poorly wilting and necrosis, while only the upper leaves of the transgenic lines exhibited brown spots or chlorosis (Figure 1B). These findings demonstrated that heterologous expression of *HIOMT* conferred improved apple plants salt stress tolerance.

To study variations in melatonin metabolism under salinity condition, we surveyed the transcript levels of four core melatonin synthesis genes. Under 1/2 nutrient solution culture condition, their expression levels were low. Under salinity conditions, all melatonin-related genes were induced, and the levels in transgenic lines were higher than those in the WT significantly (Figure 1C). We also measured melatonin concentration by LC-MS and found that it was significantly lower in WT plants in than in transgenic lines under normal and salinity conditions. The melatonin concentration upregulated by salinity and was highest in the H5 lines, reaching 1.81 ng g^−1^ FW (Figure 1D). These findings showed that heterologous expression of *HIOMT* enhanced remarkably increases in the endogenous melatonin concentration of apple leaves under salinity condition.

### 2.2. Heterologous Expression of HIOMT Promoted Better Growth and Development of Plants under Salinity Stress

After salt treatment, the shoot height (SH) did not differ significantly among lines, but there were differences in root length (RL), total fresh weight (TFW), and total dry weight (TDW). RL increased under salt stress, and transgenic lines had greater RL than the WT. We also measured leaf number (LN), leaf fresh weight (LFW), and leaf dry weight (LDW) (Table 1). Transgenic lines continued to grow new leaves under salt stress, and their LNs were higher than that of the WT; this explains why total plant weight was lower in WT that in the transgenic lines. In comparation with control conditions, the TFW of WT plants after salinity treatment decreased to 47.9%, whereas that of H1 decreased to 65.9%, that of H2 decreased to 68.2%, and that of H5 decreased to 65.0% (Table 1). These findings illustrated that heterologous expression of *HIOMT* may effectively moderate some of the defective influences of salinity on the growth of apple lines.

### 2.3. Heterologous Expression of HIOMT Changed REL, Root Vitality, and MDA Concentration in Apple

**Figure 2:**
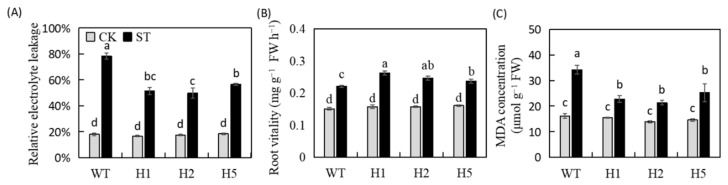
Heterologous expression of *HIOMT* had an impact on REL, Root vitality and MDA concentration under control and salinity conditions. (**A**) Leaf relative electrolyte leakage (REL); (**B**) root vitality and (**C**) leaf MDA concentration. Values are noted as means of 3 replicates ± SE. Based on Tukey’ s multi-range test (*p* < 0.05), not the same lowercase letters were used to represent statistically significant differences.

Plant abiotic stress tolerance be assessed by basic physiological indicators such as relative electrolyte leakage rate (REL), root vitality and MDA concentration. No clearly differences in the three indicators among the control lines. After salt stress, REL was 21.76–28.41% lower in transgenic lines than in WT (Figure 2A). Root vitality increased, and transgenic lines showed a greater increase (Figure 2B). Finally, the MDA concentration of the WT was higher than that of the transgenic lines significantly, which was 1.11-fold that of the unstressed control (Figure 2C). These results showed that heterologous expression of *HIOMT* could improve the endurance of apple plants to salinity.

### 2.4. Heterologous Expression of HIOMT Enhanced the Antioxidant Activity of Apple Plants under Salinity Stress

**Figure 3:**
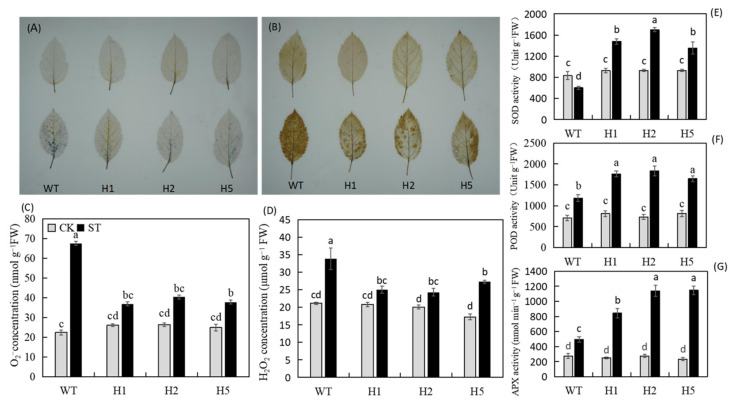
Accumulation of active oxygen and antioxidant enzyme in *HIOMT* transgenic and WT plants under control and salinity conditions. (**A**,**B**) Chemical staining of O_2_^−^ and H_2_O_2_ in transgenic lines and WT apple leaves under control and salinity condition; (**C**,**D**) O_2_^−^ and H_2_O_2_ concentrations in apple leaves with and without salinity treatment; the activity of (**E**) SOD; (**F**) POD and (**G**) APX in transgenic Lines and WT apple leaves under control and salinity condition. Values are noted as means of 3 replicates ± SE. Based on Tukey’ s multi-range test (*p* < 0.05), not the same lowercase letters were used to represent statistically significant differences.

According to the histochemical staining results after salt stress, the WT apple leaves showed darker blue and yellow colors after staining with 3,3′-diaminobenzidine (NBT) and nitro blue tetrazolium (DAB) (Figure 3A,B). We further confirmed it by quantitative analysis. The transgenic lines accumulated significantly fewer ROS than the WT (Figure 3C,D). In addition, after salt treatment, the SOD, POD, and APX activities of all plants were significantly upregulated and were higher in the transgenic lines than in the WT obviously (Figure 3E–G). Therefore, under salinity condition, heterologous expression of *HIOMT* enhanced the activity of antioxidant enzymes, enabling apple plants to avoid excessive ROS accumulation.

### 2.5. Heterologous Expression of HIOMT Enabled Apple Plants to Maintain Higher Photosynthetic Capacity under Salinity Stress

**Figure 4:**
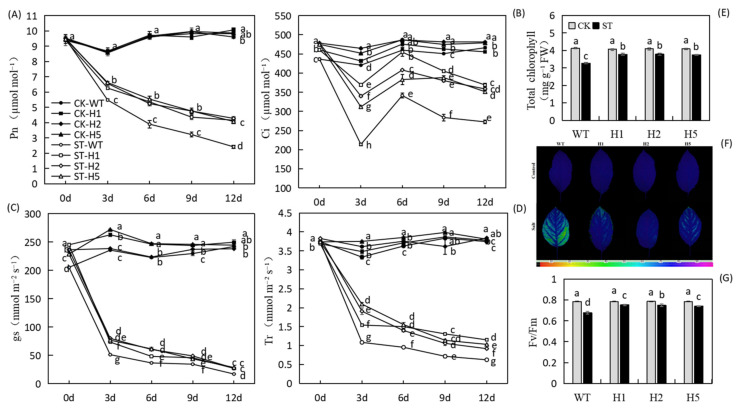
Influences in photosynthetic capacity of heterologous expression of *HIOMT* in apple under control and salinity conditions. (**A**) Changes in Pn; (**B**) gs; (**C**) Ci and (**D**) Tr; (**E**) the concentration of total chlorophyll; (**F**) chlorophyll fluorescence images; (**G**) Fv/Fm ratios of WT and transgenic lines treated with and without salinity. Values are noted as means of 5 replicates ± SE. Based on Tukey’ s multi-range test (*p* < 0.05), not the same lowercase letters were used to represent statistically significant differences.

We measured the photosynthetic parameters of the plants every three days throughout the entire salinity stress process. Under control conditions, no significant differences among the genotypes in Pn (net photosynthetic rate), Ci (intercellular CO_2_ concentration), gs (stomatal conductance), or Tr (transpiration rate). Under salt treatment, all four parameters had decreased rapidly in all lines by the third day, but those of the WT had decreased more (Figure 4A–D). The downward trend slowed after day 3, but the values of all parameters were significantly lower in the WT than in the transgenic plants. The Pn of the transgenic plants was about 1.7 times that of the WT. Total chlorophyll concentration also reduced in answer to salt stress, but *HIOMT* heterologous expression alleviated this decrease (Figure 4E).

Photosystem II (PSII) is considered to be the primary site of photoinhibition in plants. We measured its maximum photochemical efficiency (Fv/Fm) and found that under salinity condition, the Fv/Fm values of the three transgenic lines decreased by about 3.9%, 4.5%, and 5.6%, whereas that of the WT was reduced to 0.6774, a decrease of 13.6% (Figure 4F,G). These data show that the heterologous expression of *HIOMT* could alleviate damage to plant photosynthetic capacity under salt treatment and enhance plant salt tolerance to some extent.

### 2.6. Heterologous Expression of HIOMT Alleviated Stomatal Closure in Apple under Salinity Stress

**Figure 5:**
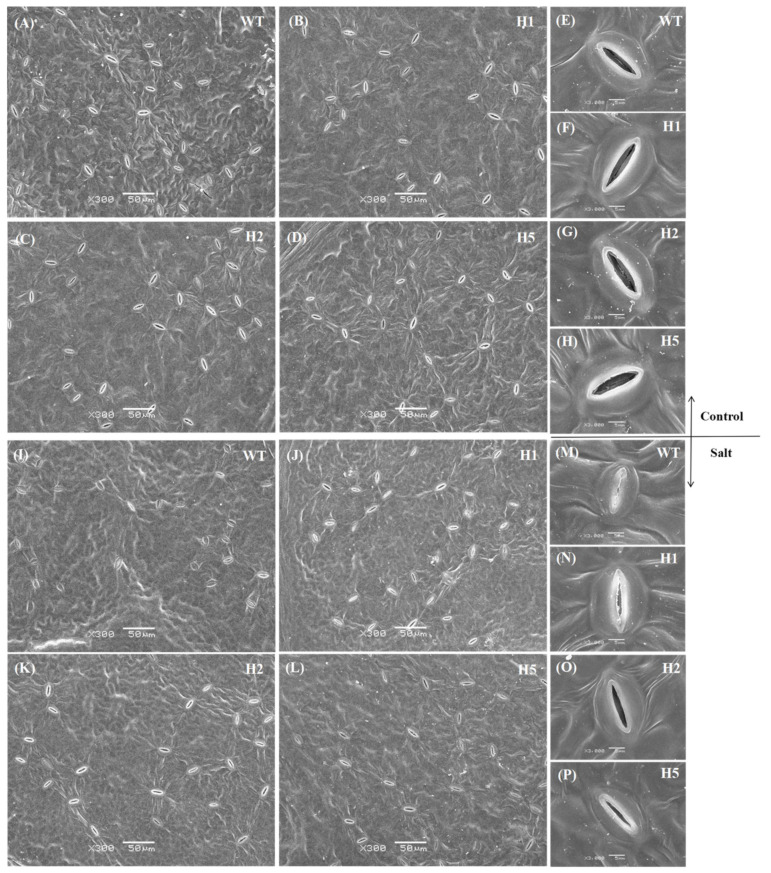
Changes in stomatal morphology, scanning electron microscopy (SEM) images of stomata from *HIOMT* transgenic and WT plants under control and salinity conditions. (**A**–**H**) Control, changes in stomatal morphology of WT, H1, H2, H5 under control condition, (**A**–**D**) magnification 300×, scale bars = 50 μm; (**E**–**H**) magnification 3000×, scale bars = 5 μm. (**I**–**P**) Salt, changes in stomatal morphology of WT, H1, H2, H5 under salt condition, (**I**–**L**) magnification 300×, scale bars = 50 μm; (**M**–**P**) magnification 3000×, scale bars = 5 μm.

**Figure 6:**
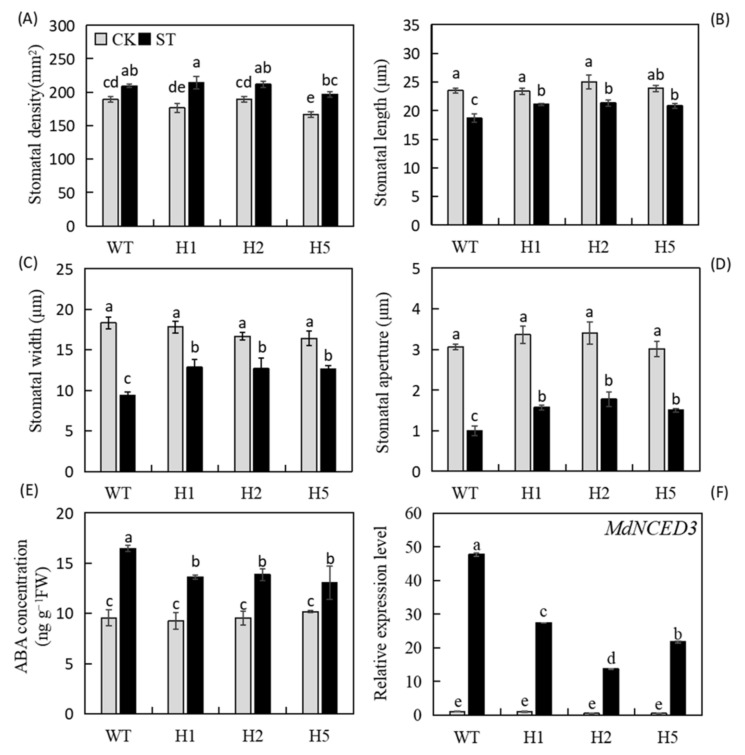
Changes in ABA concentration and ABA synthesis genes expression of WT and *HIOMT* plants under control and salinity conditions. Changes in (**A**) stomatal density, (**B**) stomatal length, (**C**) stomatal width and (**D**) stomatal aperture under salt stress. (**E**) the concentration of ABA; Changes in expression of (**F**) *MdNCED3*. Values are noted as means of 3 replicates ± SE. Based on Tukey’ s multi-range test (*p* < 0.05), not the same lowercase letters were used to represent statistically significant differences.

Plant stomata undergo a series of changes under salt stress. We found that stomatal length, width, and aperture of apple leaves decreased after salt treatment compared with normal conditions. Stomatal contraction was most obvious in the WT (Figure 5 and Figure 6A–D). The stomatal density of all lines increased under salt stress relative to normal conditions, and there were no distinct differences among the lines. ABA mediates the response of stomatal aperture to changes in the external environment. We measured leaf ABA concentration after salt treatment and found that under normal conditions, little difference in ABA concentration among genotypes. The ABA concentration increased in all lines after salt treatment, but this increase was greatest in the leaves of WT plants; their ABA levels were 1.2 times those of the transgenic plants (Figure 6F). We measured the transcript levels of *MdNCED3*, a key gene that induces ABA biosynthesis under stress. We found that salt treatment significantly increased its expression, but its expression was significantly lower in transgenic lines than in the WT (Figure 6G). These findings indicated that heterologous expression of *HIOMT* could alleviate the ABA concentration increasement in and the decrease in stomatal aperture under salinity condition.

### 2.7. Heterologous Expression of HIOMT Reduced the Na^+^/K^+^ Ratio in Apple Plants under Salinity Stress

**Figure 7:**
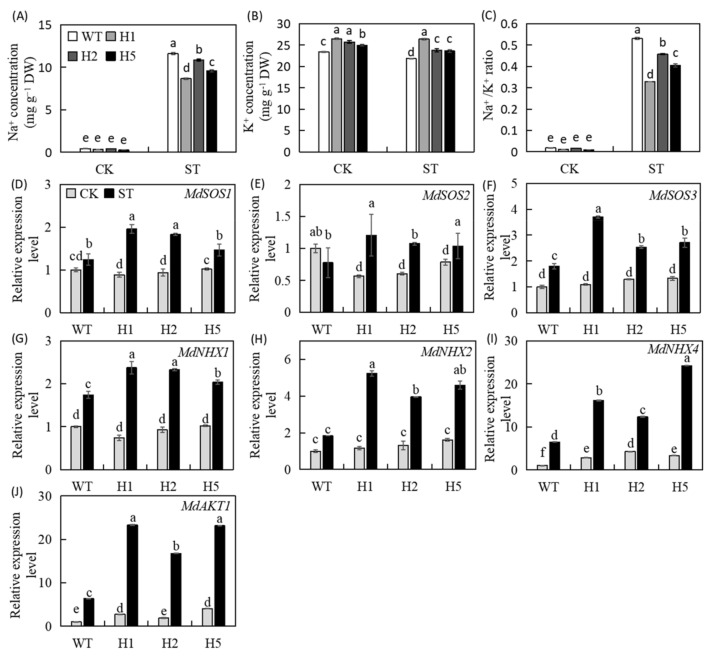
The changes of Na^+^ and K^+^ of WT and *HIOMT* plants leaves and the expression of salt-related genes of WT and *HIOMT* plants leaves under control and salinity conditions. (**A**) Changes in concentrations of Na^+^; (**B**) concentrations of K^+^ and (**C**) Na^+^/K^+^ ratios in the leaves of WT and transgenic lines. Changes in expression of (**D**) *MdSOS1*; (**E**) *MdSOS2*; (**F**) *MdSOS3*; (**G**) *MdNHX1*; (**H**) *MdNHX2*; (**I**) *MdNHX4*; (**J**) *MdAKT1*; Values are noted as means of 3 replicates ± SE. Based on Tukey’ s multi-range test (*p* < 0.05), not the same lowercase letters were used to represent statistically significant differences.

Under normal conditions, levels of Na^+^ ions were very low in the leaves of transgenic and WT plants, and no significant differences among the lines. The K^+^ ion concentration was 23.4–26.5 mg g^−1^ DW. Plants were under NaCl stress for 15 d, the Na^+^ ion concentration of all plant leaves increased significantly, but that of the three transgenic lines was significantly lower (74.8%, 93.7%, and 82.5%) than that of the WT. The K^+^ ion concentration declined over time, and the final Na^+^/K^+^ ratio of the WT was much higher than that of the transgenic lines (Figure 7A–C). We selected key salt stress–related genes for quantitative analysis, and we found that the expression levels of *MdSOS1*, *MdSOS2*, and *MdSOS3* in the SOS pathway were all upregulated in leaves under salinity condition (Figure 7D–F). The expression levels of *MdNHX1*, *MdNHX2*, and *MdNHX4* also showed the same trend (Figure 7G–I). At the same time, the expression of a membrane protein involved in K^+^ ion transport in leaves (AKT potassium transport protein) was also significantly upregulated (Figure 7J). These results showed that heterologous expression of *HIOMT* stabilized the leaf Na^+^/K^+^ ratio under salinity condition.

### 2.8. Heterologous Expression of HIOMT Mediated Amino Acid Metabolism under Salinity Stress

**Figure 8:**
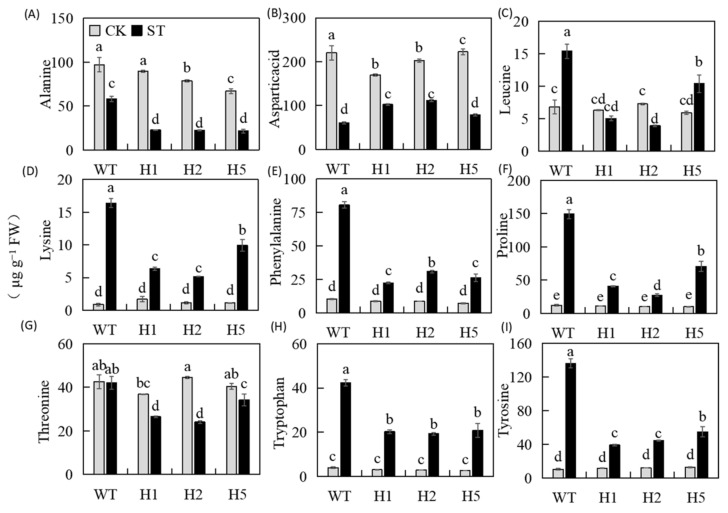
Changes in the concentration of amino acids in the leaves of WT and *HIOMT* plants after salt stress. The concentration of (**A**) Alanine, (**B**) Aspartic acid, (**C**) Leucine, (**D**) Lysine, (**E**) Phenylalanine, (**F**) Proline, (**G**) Threonine, (**H**) Tryptophan, (**I**) Tyrosine. Values are noted as means of 3 replicates ± SE. Based on Tukey’ s multi-range test (*p* < 0.05), not the same lowercase letters were used to represent statistically significant differences.

Under control conditions, there were no clear diversities in the concentration of amino acid between the lines (Figure 8), but there were significant differences between the transgenic and WT plants under salt stress. The concentrations of alanine (Ala), aspartic acid (Asp), and threonine (Thr) were lower after stress than under control conditions. The concentration of Ala and Thr in the transgenic lines had dropped more, whereas the concentration of Asp was slightly higher in the transgenic lines than in the WT (Figure 8A,B,G). After stress, the concentration of most amino acids increased; these included lysine (Lys), phenylalanine (Phe), proline (Pro), tyrosine (Thr), and tryptophan (Tyr). Their concentration increased more in the WT and were 18.5, 7.8, 12.4, 13.1, and 10.9 times those of the unstressed control; proline concentration increased to 149.4 μg/g. The amino acid concentration of the transgenic lines were lower than those of the WT significantly (Figure 8D–F,H,I). The leucine (Leu) concentration of the WT and H5 was higher after stress than in the unstressed control; the Leu concentration of H5 increased less, the Leu concentration of H1 and H2 was reduced, and the Leu concentration of WT was the highest (Figure 8C). These results showed that heterologous expression of *HIOMT* in apples altered amino acid metabolism under salt stress.

## 3. Discussion

There is abundance evidence conducted studies the important roles of melatonin in plant tolerance to stressors [23,27,28]. HIOMT is an enzyme that has a rate-limiting effect in the process of human melatonin synthesis. The genes encoding HIOMT enzymes was transferred into GL-3 apple, which increased endogenous melatonin concentration in plants significantly [31]. The ability of exogenous melatonin to improve *Malus hupehensis* endurance to salinity has been reported previously [24]. However, barely known about the specific function of *HIOMT* in the apple salinity response. At this respect, we discovered that heterologous expression of *HIOMT* induced melatonin-related genes up-regulated under salt stress (Figure 1A,C), and we then used hydroponic salt treatment to understand of the mechanisms by which HIOMT functions in response to salinity stress.

We discovered that the melatonin concentration enhanced in all lines under salinity condition, but it was much lower in WT than in transgenic lines under both normal and salinity conditions (Figure 1D). This result is in accordance with antecedent research showing that changes in the external environment cause plants to activate the endogenous melatonin regulatory pathway and change their hormone levels to improve stress tolerance under various stresses, including salt stress [32,33,34]. A number of other studies have also confirmed that transgenic plants with foreign genes can exhibit increased melatonin production and improved stress tolerance [2,35]. Basic salt stress phenotypes include plant growth inhibition, dysplasia, metabolic disorders, and ion toxicity [36]. The clearest manifestation of salt stress is chlorosis and wilting of leaves (Figure 1B). Electrical conductivity and MDA concentration can also reflect the severity of stress damage [1]. These two indicators were lower in the transgenic lines than in the WT significantly (Figure 2A,C), and transgenic lines showed higher levels of growth, root activity, and other physiological indicators (Table 1 and Figure 2B). These findings show that the heterologous expression of *HIOMT* increased melatonin concentration, reduced the inhibitory effect of salinity on plant development, and alleviated the symptoms of salt damage.

Salinity leads to damage for the photosynthetic system, inhibits photosynthetic electron transfer, and promotes excessive ROS accumulation [11]. The antioxidant enzyme system has vital function in removing excess ROS from plant tissues [10]. Chen et al. [37] discovered that exogenous melatonin improved the activity of antioxidant enzymes in corn seeds under salinity condition, thereby increasing the salt tolerance of corn. They also discovered that after salinity stress, the concentration of H_2_O_2_ and O_2_^−^ was lower in apple leaves that overexpressed *HIOMT* than in the WT, and the activities of the ROS scavenging enzymes SOD, POD, and APX were significantly higher (Figure 3A–G). These results indicated that *HIOMT* heterologous expression could increase the antioxidant enzyme activity in apple leaves, removing excess ROS and preventing plants from oxidative damage under salt stress.

Reductions in Pn under salt stress are related to changes in leaf anatomical characteristics [5]. The combination of stomatal closure, chlorophyll loss, and other metabolic changes may lead to a decline in photosynthetic capacity [2]. Here, we found that *HIOMT* heterologous expression reduced stomatal contraction (Figure 5A–P and Figure 6A–D) and total chlorophyll loss under salt stress (Figure 4E), and this may interpret the stronger photosynthetic capacity of the transgenic lines (Figure 4A–D). Some degree of stomatal closure under salt stress is a self-protective mechanism [38]. In a high-salt environment, stomatal closure prevents the water lost through transpiration from exceeding the water absorbed by the roots, thereby preventing dehydration. An increase in stomatal density enhances the capacity for CO_2_ assimilation and improves the stress resistance of plants [39]. Reductions in chlorophyll concentration and chlorophyll fluorescence parameters occur simultaneously, and chlorophyll fluorescence can provide useful information on changes in photosynthetic performance under stress [40]. Here, we found that transgenic lines also had higher Fv/Fm data than the WT under salt stress (Figure 4F,G). Wei et al. [41] proved that exogenous melatonin appliance helped apple leaves to maintain a higher Fv/Fm value when subjected to photooxidative stress, a result consistent with our present findings.

ABA is closely related to plant stress response [42]. ABA accumulation can cause stomatal closure to prevent water loss while also mediating related genes to facilitate leaf osmotic regulation [43]. To explore stomatal function under salt stress, we measured leaf ABA concentration and found that it was consistent with the changes in stomatal aperture of each line (Figure 6E,F), suggesting that heterologous expression of *HIOMT* can alter the plant’s endogenous ABA concentration to control changes in stomatal behavior. Sato et al. [44] reported that *NCED3* may be a key enzyme that promotes ABA biosynthesis in Arabidopsis. Our results show that *MdNCED3* was significantly upregulated under salt stress, but heterologous expression of *HIOMT* inhibited its expression, compliance with the discoveries of Li et al. [45], in which exogenous melatonin restrained the expression of the ABA synthesis gene *MdNCED3* under drought.

Under salinity condition, the absorption and regulation of Na^+^ and K^+^ ions is an essential ingredient of plant salt tolerance [17,46]. Here, we gauged the concentrations of Na^+^ and K^+^ in the leaves and found that the transgenic lines had strong salt tolerance (Figure 7A–C). This is related to their restriction of Na^+^ absorption, stable K^+^ concentration, and maintenance of Na^+^/K^+^ balance under salinity condition [24]. The SOS pathway uses the driving force of the plasma membrane proton gradient to discharge excess Na^+^, thereby reducing the toxic effect of intracellular Na^+^ [47]. The NHX pathway acts mainly in concert with the H^+^-ATPase and H^+^-PPase proton pumps on the tonoplast membrane, sequestering Na^+^ from the cytoplasm into the vacuole and thereby reducing its potential toxicity [48]. Here, we discovered that salt treatment improved the expression of *SOS* genes apple plants leaves (Figure 7D–F). The expression of *NHX* genes showed a similar trend in all lines (Figure 7G–I), indicating that the Na^+^ efflux ability of the transgenic lines was stronger, enabling them to effectively alleviate the accumulation of Na^+^ in leaves under salinity condition. AKT1 is an internal K^+^ ion current channel, and overexpression of *SsAKT1* in *Suaeda salsa* promoted the accumulation of potassium ions under salinity condition and enhanced plant salt endurance [49]. Therefore, we also measured the expression levels of *MdAKT1* genes and found that their upregulation under salt stress was higher in transgenic lines than in the WT (Figure 7J). Based on these results, we hypothesize that the heterologous expression of *HIOMT* helps to maintain Na^+^/K^+^ homeostasis by mediating the expression of genes connected with ion transport, thereby alleviating symptoms of leaf salt damage. The specific regulatory mechanism requires further exploration.

As the basic unit of biologically functional proteins, amino acids have vital functions in plant signal transduction and stress resistance [50]. Here, we found that the increase in Pro under salinity condition was much more in the WT than in the transgenic lines (Figure 8F). This result is consistent with the review by Mansour and Ali [51], who reported that Pro overproduction is positively correlated with the salt stress pressure experienced by plants. For example, Kanawapee et al. [52] obviously showed that the proline accumulation under salt stress showed a trend opposite to that of salt tolerance level and was consistent with changes in the Na^+^/K^+^ ratio. Here, we suspect that the slight increase in Pro concentration in transgenic lines under salt stress occurred because the 120 mM salt concentration caused a lighter stress, and the transgenic plants were able to adjust their osmotic potential, whereas the WT could not. In addition to proline, we also focused on three AAAs (Trp, Tyr, and Phe) whose concentration showed consistent changes with salt stress (Figure 8E,H,I). Studies have shown that diverse secondary metabolites are derived from these AAAs [22]. The synthesis of auxins and alkaloids requires Trp as a precursor material, Tyr is the precursor of betaine, and anthocyanins, flavonoids, and others are derivatives of Phe; all of these secondary metabolites play important roles in protecting plants from stress [53]. Trp, a precursor for melatonin synthesis, can be converted to tryptamine via TDC to synthesize melatonin [54]. We found that the expression level of *MdTDC1* was much higher in transgenic lines than in the WT (Figure 1B), while the Trp concentration of them was lower than WT (Figure 8H). This result may be due to Trp in the transgenic lines be converted to melatonin by *MdTDC1* and other melatonin synthases, thereby further improving the resistance of transgenic lines to salt stress. In summary, heterologous expression of *HIOMT* can directly or indirectly mediate amino acid metabolism to increase plant stress adaptability.

## 4. Materials and Methods

### 4.1. Plant Materials and Treatments

Tissue-cultured apple plants of *M. domestica* ‘GL-3’ lines (WT) and transgenic GL-3 plants (heterologous expression *HIOMT* lines) were acquired from the study in our laboratory previously, the heterologous expression levels of *HIOMT* (Accession#M83779) lines were increased by 52.06, 203.05 and 90.92-fold in the H1, H2 and H5 lines, respectively [31]. After 30 d of subculturing and 40 d of rooting, tissue culture seedlings were transplanted to 12 × 12 cm^2^ nutrition bowls filled with the mixture of soil, vermiculite, perlite (v1:v2:v3 = 4:1:1). The bowls were placed in a constant temperature light incubator. After the seedlings had grown about 7–8 fully expanded true leaves, they were moved to hydroponic containers (35 × 28 × 15 cm^3^) that were wrapped in black plastic and contained 6.5 L half concentration Hoagland’s nutrient solution. The pH of the nutrient solution was adjusted to 6.0 ± 0.2 with H_3_PO_4_, and it was changed every 5 d. The culture system was designed and improved according to Li et al. previous experiments [24]. The experiments performed in a hydroponic laboratory in Northwest A & F University Yangling (34°20′ N, 108°24′ E), Shaanxi Province, China. Plants growth with condition included a 14 h photoperiod (the light intensity was 160 µmol m^−2^·s^−1^), 24 ± 2 °C/16 ± 2 °C day/night and 60 ± 5% relative humidity [55]. After 2 weeks of pre-adaptation, plants of consistent size and healthy were picked for treatment. The seedlings were divided into two groups, each containing 45 plants: (1) the control group (CK) was grown in half concentration Hoagland nutrient solution as the control, and (2) the salt stress group (ST) was grown in half concentration Hoagland nutrient solution supplemented with 120 mM NaCl. Both group with a pH of 6.5 ± 0.2. The nutrient solution was updated at an interval of 3 d and treated it for 15 d.

After salt application, control and treated plant leaf tips were taken for *MdASMT* expression analysis. At the end of the treatment, the 4th to 6th leaves of the shoot tips were collected from 12 plants per line and stored at −80 °C for subsequent index determination.

### 4.2. Determination of Melatonin

The extraction steps of melatonin from leaves referred to the description of Pothinuch and Tongchitpakdee [56]. In brief, a 0.3-g frozen leaf tissue was weighed, each treatment was set up with 5 biological replicates. 5 mL methanol was added; the mixture was sonicated at 4 °C and 40 Hz for 40 min. The centrifuge was set to 10,000 g and 4 °C for 15 min of centrifugation. All the supernatant was collected, placed in a new centrifuge tube, and dried under nitrogen. After reconstitution in pure methanol, the melatonin concentration in the reconstituted solution was determined by high performance liquid chromatography tandem mass spectrometry (HPLC-MS/MS), the above steps refer to Zhao et al. [57]. Three analytical replicates were selected from the measurement results for analysis.

### 4.3. Growth Measurements

Six plants of each genotype were selected for growth measurements after 15 d of hydroponic treatment. The height from the junction of the rhizome to the top bud of the plant was measured with an iron ruler; the leaves number was accurately counted; and the fresh and dry weights of each plant were recorded [58]. The formula for calculating the relative growth rate was as follows: the average weight of the treated plants divided by the average weight of the control plants.

### 4.4. Measurement of Relative Electrolyte Leakage, Root Vitality, and MDA Concentration

The REL of control leaves, plant leaves under salt stress, and double distilled water were recorded as S1, S2, S0, respectively, refer to the methods of Dionisio-Sese and Tobita for relative conductivity measurement, and calculate REL = S1−S0/S2−S0 × 100%. [59]. After 15 d, white roots were harvested from 5 individual plants and stained with triphenyltetrazolium chloride as described by Gong [34]. Malondialdehyde (MDA) concentration was measured using a test kit (Suzhou Coming Biotechnology) according to the instructions of manufacturer. Three analytical replicates were selected from the measurement results for analysis.

### 4.5. Qualitative and Quantitative Determination of H_2_O_2_ and O_2_^−^

At the end of the treatment, 5–8 fully developed mature leaves were obtained from 5 plants per line for histochemical staining with DAB and NBT. Tissue was decolorized thoroughly to observe the accumulation of H_2_O_2_ and O_2_^−^ as described previously [60]. Quantitative analysis of each concentration was performed with a test kit (Suzhou Comin Biotechnology). Three analytical replicates were selected from the measurement results for analysis.

### 4.6. Determination of Antioxidant Enzyme Activity

After 15 days of treatment, the frozen sample of the leaves was ground into powder, weighed 0.1 g into a 2 mL centrifuge tube, each treatment was set up with 3 biological replicates. Then used a test kit (Suzhou Comin Biotechnology) according to the instructions of manufacturer to determine the enzyme activities of SOD, POD, and APX, three replicates for each treatment [61]. Three analytical replicates were selected from the measurement results for analysis.

### 4.7. Quantification of Photosynthetic Parameters

During the whole process of salt treatment, every three days we measured Pn, Ci, gs, and Tr using a CIRAS-3 portable photosynthesis system (CIRAS-3, PP Systems, Amesbury, MA, USA) between 9:00 and 11:00. The instrument parameter setting referred to the description of Liu et al. [55]. For each treatment, 5–8 functional leaves at the same position are selected for the measurement of photosynthetic parameters. Five analytical replicates were selected from the measurement results for analysis.

### 4.8. Chlorophyll Concentration and Fv/Fm Measurements

Chlorophyll concentration was measured using the method of Arnon [62]. In brief, 8 mL 80% acetone was added to 0.1 g fresh leaves in the dark for at least 24 h to extract pigments, and the mixture was shaken 3–4 times during this period until the leaves turned white. Each treatment was set up with 3 biological replicates. The optical density values were measured at 663, 645, and 470 nm with a UV-2250 spectrophotometer (Shimadzu, Kyoto, Japan).

To measure chlorophyll fluorescence, fully expanded leaves with minimal salt damage from the same position on 5 plants were wrapped in tin foil. After 20 min of dark adaptation, the leaves were cut and placed into the chlorophyll fluorescence imaging system (IMAGING-PAM, Heinz Walz, Effeltrich, Germany) to monitor the maximum quantum efficiency of photosystem II (Fv/Fm). The parameters of the imaging system were set as follows: meas. light 3, act. light 5, ext. light 3, int 10, and FoFm 6 [63]. Five analytical replicates were selected from the measurement results for analysis.

### 4.9. Stomatal Observations by Scanning Electron Microscopy (SEM)

The measurement of stomatal characteristics refered to Bai et al. [64]. After treatment, three upper leaves were cut at the same leaf position for each treatment, avoiding the main and lateral veins; the leaves were cut into 5 mm × 5 mm squares, stored in pre-cooled 25% glutaraldehyde prepared in advance with 0.2 M phosphate buffered saline (PBS) (pH 7.4). The vacuum was applied with a syringe until the leaf sunk to the bottom of the liquid. The sample was wrapped with tin foil and placed in the refrigerator at 4 °C. It was then washed with PBS (pH 6.8), dehydrated with an ethanol gradient, and placed in 1 mL of isoamyl acetate. After drying and spraying with gold, leaf stomata were discerned and photographed with the scanning electron microscope at 300 and 3000 magnifications. Five pictures were taken for each treatment at different magnifications. Finally, the stomatal density, size, and degree of opening were analyzed using ImageJ software.

### 4.10. Determination of ABA Concentration

Determination of ABA concentration based on the description by Zhang et al. [65]. In brief, endogenous ABA was extracted using extraction liquid (methanol: isopropanol: acetic acid = 20:79:1). A 0.1-g frozen tissue was combined with 1 mL of extraction liquid, vortexed for 5 min, and then put in refrigerator at −20 °C for 12 h. The centrifuge was set to 4 °C and 12,000 rpm for 10 min of centrifugation. All the supernatant was aspirated and filtered into a sample bottle with a 0.22-μm filter, each treatment was set up with 3 biological replicates, and the ABA concentration of the supernatant was measured by HPLC-MS/MS. Three analytical replicates were selected from the measurement results for analysis.

### 4.11. Measurements of Sodium and Potassium Ions

At the end of the stress proceeding, leaves from 10 plants were randomly picked out for each treatment and washed thoroughly. The leaves were wiped dry with folded blotting paper. Samples were dried at 105 °C for 20 min and then transferred to a 65 °C oven for at least 72 h. After the dried tissue was ground with a mortar and a tissue lyser, it was stored in a 10-mL centrifuge tube for determination of mineral element concentration after sieving by flame spectroscopy (M410; Sherwood Scientific, Cambridge, UK). The specific steps wrote in Liang et al. [58]. Three analytical replicates were selected from the measurement results for analysis.

### 4.12. Measurements of Amino Acids

Amino acids were extracted and the concentration of them were measured as accounted by Huo et al. [61] with improvements. In brief, 0.1 g of frozen leaf sample was weighed, soaked in 1 mL 50% ethanol, shaken at 4 °C for 5 min, the centrifuge was set to 12,000 rpm, and the time was 10 min, each treatment was set up with 3 biological replicates. Impurities were filtered out, the supernatant was diluted with methanol, and stored in a sample bottle. Amino acids in the sample were measured by liquid chromatography-mass spectrometry (LC-MS, LC: AC, Exion LC; MS: QTRAP 5500, AB SCIEX), and standard curves were used to calculate amino acid concentration. Three analytical replicates were selected from the measurement results for analysis.

### 4.13. qRT–PCR Analysis

Samples were thoroughly ground in liquid nitrogen. Roughly 0.05 g of the resulting powder was weighed for RNA extraction with the Wolact Plant RNA Extraction Kit (Wickband, Hong Kong, China) according to the instructions. Then the synthesize of cDNA by the Prime Script RT reagent Kit with gDNA Eraser (Perfect Real Time). The cDNA was uniformly diluted to 200 ng/μL, and qRT-PCR quantitative analysis was performed. Each gene set three biological replicates, and each replicate used 10 μL SYBR Premix Ex Taq (TaKaRa). The ΔCt value was calculated using *EF* as the internal reference gene [66]. Primer sequences are listed in Table S1. At 0, 3, 6, 9, and 12 h and 3, 6, 9, 12 and 15 d after the initiation of salt stress, the top leaves of the plants were randomly selected for measurement. Three analytical replicates were selected from the measurement results for analysis.

### 4.14. Statistical Analysis

All values were statistically analyzed using SPSS 25.0 software, and Tukey’s test was used for multiple comparisons (*p* < 0.05). Results are represented as mean ± SE.

## 5. Conclusions

**Figure 9:**
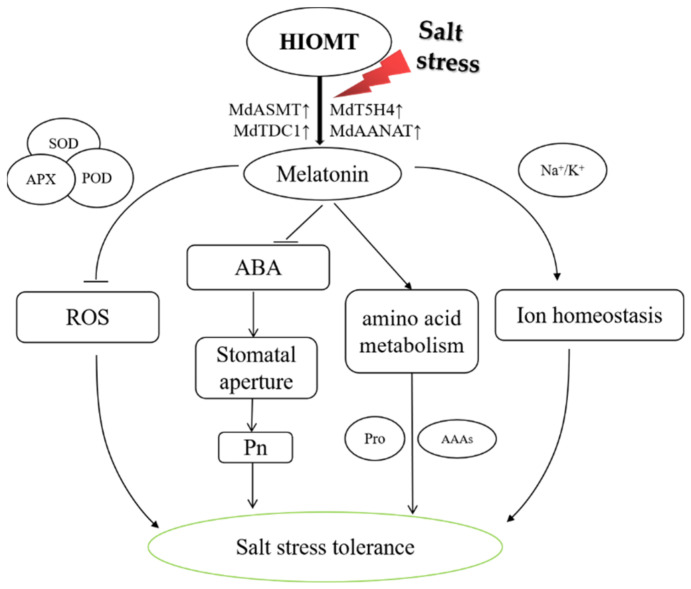
A scheduled model for accounting regulator function of *HIOMT* in physiological reaction of salinity in apple. Under salinity stress, heterologous expression of *HIOMT* enhanced the activity of antioxidant enzymes and inhibited ROS production; inhibited the increase of ABA, maintained stomatal aperture, and improved photosynthetic capacity; mediated amino acid metabolism; stabilized the balance of Na^+^, K^+^ and maintains ion homeostasis in cells. These were all through the heterologous expression of *HIOMT* resulted in an increase in endogenous melatonin. Therefore, heterologous expression of *HIOMT* improved the salt tolerance in transgenic apple plants.

Here, we showed that heterologous expression of *HIOMT* enhanced the tolerance of apple leaves to salt stress (Figure 9). Heterologous expression of *HIOMT* increased the expression of melatonin synthase genes under salinity condition and promoted an increase in endogenous melatonin concentration. *HIOMT*-mediated resistance to salinity was associated with reductions in ROS accumulation, maintenance of a strong photosynthetic capacity, regulation of osmotic pressure, and stabilization of the sodium–potassium balance. The heterologous expression of *HIOMT* increased the endogenous melatonin concentration, thereby improving the activity of antioxidant enzymes and inhibiting ROS accumulation. In addition, stomatal aperture could be adjusted by regulating ABA metabolism to restrain water loss and enhance carbon dioxide absorption, thereby improving plant photosynthetic capacity. *HIOMT* heterologous expression also helped to maintain the dynamic balance of intracellular ions by balancing the ratio of sodium and potassium ions and altering amino acid metabolism to alleviate osmotic stress. Our research provides evidence for melatonin-mediated salt tolerance and has crucial applications for improving the growth of horticultural crops on salinity soils.

## Figures and Tables

**Table 1:** Stem height (SH), root length (RL), leaf number (LN), leaf fresh weight (LFW), leaf dry weight (LDW), total fresh weight (TFW), total dry weight (TDW), relative growth of heterologous expression of *HIOMT* apple plants after 15 d under control, and salinity conditions.

Treatment	SH (cm)	RL (cm)	LN (No. Plant^−1^)	LFW (g)	LDW (g)	TFW (g)	TDW (g)	Relative
								Growth
CK-WT	13.97 ± 0.91 ^a^	7.50 ± 1.78 ^c^	12.50 ± 1.05 ^a^	1.32 ± 0.10 ^a^	0.45 ± 0.05 ^a^	2.11 ± 0.16 ^a^	0.62 ± 0.05 ^a^	47.9%
ST-WT	9.84 ± 0.26 ^b^	8.60 ± 1.36 ^b,c^	8.17 ± 0.75 ^c^	0.51 ± 0.11 ^c^	0.21 ± 0.05 ^c^	1.01 ± 0.22 ^c^	0.31 ± 0.06 ^c^	
CK-H1	13.27 ± 1.07 ^a^	9.05 ± 0.82 ^b,c^	13.00 ± 0.89 ^a^	1.34 ± 0.16 ^a^	0.43 ± 0.07 ^a^	2.11 ± 0.14 ^a^	0.61 ± 0.08 ^a^	65.9%
ST-H1	10.27 ± 0.42 ^b^	10.86 ± 2.04 ^a^	10.00 ± 1.41 ^b^	0.82 ± 0.21 ^b^	0.32 ± 0.08 ^b^	1.39 ± 0.22 ^b^	0.45 ± 0.08 ^b^	
CK-H2	13.32 ± 1.28 ^a^	8.68 ± 1.39 ^b,c^	12.50 ± 1.05 ^a^	1.36 ± 0.13 ^a^	0.48 ± 0.06 ^a^	2.20 ± 0.25 ^a^	0.64 ± 0.08 ^a^	68.2%
ST-H2	9.97 ± 0.33 ^b^	10.30 ± 0.97 ^a,b^	9.83 ± 1.47 ^b^	0.89 ± 0.13 ^b^	0.35 ± 0.07 ^b^	1.50 ± 0.15 ^b^	0.48 ± 0.08 ^b^	
CK-H5	13.90 ± 1.45 ^a^	8.48 ± 1.53 ^b,c^	13.67 ± 1.63 ^a^	1.40 ± 0.17 ^a^	0.47 ± 0.06 ^a^	2.26 ± 0.24 ^a^	0.64 ± 0.08 ^a^	65.0%
ST-H5	10.41 ± 0.56 ^b^	9.67 ± 0.53 ^a, b^	9.50 ± 1.05 ^b^	0.85 ± 0.12 ^b^	0.34 ± 0.07 ^b^	1.47 ± 0.19 ^b^	0.48 ± 0.09 ^b^	

Values are noted as means of 6 replicates ± SE. Based on Tukey’ s multi-range test (*p* < 0.05), not the same lowercase letters were used to represent statistically significant differences.

## Data Availability

Not applicable.

## Supplementary Materials

The following are available online at https://www.mdpi.com/article/10.3390/ijms222212425/s1, Table S1: Primers used in this study.
