# Supplementary material for: Heterologous Expression of the Melatonin-Related Gene HIOMT Improves Salt Tolerance in Malus domestica
Source: Int J Mol Sci. 2021 Nov 17;22(22):12425. doi: 10.3390/ijms222212425 (PMC8620682; doi:10.3390/ijms222212425)
Supplement: Supplementary file 1 [file ijms-22-12425-s001.zip › ijms-1453734-supplementary.pdf]

Table S1 Primers used in this study.

Name	Sequence (5'-3')
EF	F: ATTCAAGTATGCCTGGGTGC
	R: CAGTCAGCCTGTGATGTTCC
MdTDC	F: TCACGCTGTGGTTGGAGGT
	R: CTGCATGCTCCTGAACCAAC
MdT5H4	F: TCGGTGACATGTTTGCTGC
	R: GGAAACCTTGGTCTGGCG
MdAANAT2	F: GAATCACCGTCCACGCTCC
	R: GAAATGCTTCCGATGTCCC
MdASMT1	F: AGAGGAGCGAGAAAGACTGGA
	R: CTAAAGAAAAACTTCAATGAGGGAT
MdSOS1	F: TCCGGTTAATCCATCACACACCGT
	R: TTTGCTGCCCTGGAGGATTTGTTG
MdSOS2	F: CAAAAGCACCATTCTCAAGCAC
	R: CCGACCAGCCAAAACCTCT
MdSOS3	F: AAGGCAAGGCGGCAGTTT
	R: GCGAGGCATTGGGATGAA
MdNHX1	F: AAGCGACAGTCCTGGAACATCAGT
	R: TATTATCACTTGCTGCCGGAGGCT
MdNHX2	F: ATGCGTGGCTCTGTTTCAAT
	R: AACTGTGATGGTGCTGGTGA
MdNHX4	F: ATCACCAAAACCACCAACCA
	R: GCCACACTTCTTAGGCAACG
MdAKT1	F: GCGGAGACGAAAAGTCCTAA
	R: AGTGGGAGCAGCACAAGTTT
